# Medical specialty selection criteria of Israeli medical students early in their clinical experience: subgroups

**DOI:** 10.1186/s13584-018-0215-2

**Published:** 2018-04-18

**Authors:** Alexander Avidan, Charles Weissman, Uriel Elchalal, Howard Tandeter, Rachel Yaffa Zisk-Rony

**Affiliations:** 1 Department of Anesthesiology and Critical Care Medicine, Hadassah – Hebrew University Medical Center, Kiryat Hadassah, POB 12000, 91120 Jerusalem, Israel; 2 Department of Family Medicine, Ben Gurion University Joyce and Irving Goldman School of Medicine, Be’er Sheva, Israel; 3 Department of Obstetrics and Gynecology, Hadassah-Hebrew University Medical Center, Hebrew University – Hadassah School of Medicine, Jerusalem, Israel; 4 Hebrew University – Hadassah Henrietta Szold School of Nursing, Jerusalem, Israel

**Keywords:** Medical students, Medical education, Residency, Medical specialty selection, Career choice

## Abstract

**Background:**

Israeli medical school classes include a number of student subgroups. Therefore, interventions aimed at recruiting medical students to the various specialties should to be tailored to each subgroup.

**Methods:**

Questionnaires, distributed to 6 consecutive 5th-year classes of the Hebrew University – Hadassah School of Medicine, elicited information on criteria for choosing a career specialty, criteria for choosing a residency program and the importance of finding a specialty interesting and challenging when choosing a residency.

**Results:**

Completed questionnaires were returned by 540 of 769 (70%) students. The decision processes for choosing a medical specialty and choosing a residency program were different. Family and colleagues had minimal influence on choosing a specialty, while family and their residential locality had much influence on choosing a residency, especially among women. Older age, marriage, and spousal influence were positively associated with choice of a specialty. Two-thirds of the students had completed military service, 20% were attending medical school prior to military service, 5% had completed national service and 9% had entered medical school without serving. Despite the pre-military subgroup being younger and having another 7 years of medical school, internship and military service before residency, they had begun thinking about which specialty to choose, just like the post-military students. When choosing a residency program, post-military women were more influenced by their families and family residential locality than their pre-military counterparts; differences ascribed to the older and often married post-military women having or wanting to begin families. This difference was reinforced by fewer post- than pre-military women willing to wait 2-3 years for a residency in the specialty that interested them most and were willing to begin residency immediately after internship in a specialty that interested them less.

**Conclusions:**

Medical school classes are composed of various subgroups, each with its own characteristics. It is important to differentiate between choosing a specialty and a residency program. Choosing a specialty is a uniquely personal decision with some spousal influence among married students. It is of central importance even among pre-military students not slated to begin residency for many years. In contrast, choosing a residency program is influenced by family, where one grew up and other family-related considerations.

## Background

Medical students’ choices of a specialty are a major determinant of the future composition of the physician workforce. Therefore, it is incumbent upon healthcare system leaders to gain insight into the selection process in order to avoid shortages and surpluses among the various specialties. Shortages of vital specialists can greatly affect the ability of the system to deliver sufficient and efficient services, while surpluses can result in service overutilization [1]. Furthermore, to achieve maximum impact, interventions aimed at recruiting medical students to the various specialties should to be timed to the period when they are actively contemplating which specialty to choose. Moreover, these interventions should be tailored to the various subgroups of students. This approach can be likened to marketing campaigns wherein a product is differentially marketed during the appropriate season to a variety of age and socioeconomic groups [2, 3].

Israeli medical school classes are composed of a number of student subgroups. One subgroup enters medical school upon completing their military obligations. Men generally serve for 3 years and are thus older than the women who serve for 2 years. Two other subgroups of students include one doing 1-2 years of national service rather than military service prior to entering medical school and another subgroup composed of Israeli Arabs that does not perform either military or national service, thus beginning medical school immediately upon graduating high school. A fourth student subgroup is admitted to medical school upon completing high school, deferring military service until after their internship at which time they have a 5-year military obligation. These students, along with those who did not perform military service, tend to be younger in age than those in the other two groups. A major aim of the present study was to examine the differences between the criteria each of these subgroups uses for choosing medical specialties and residency programs. Furthermore, the heterogeneous population of the medical school classes provided the opportunity to examine the influence of age on attitudes toward specialty and residency program selection.

The present study is a follow-up to one that examined the criteria students early in their clinical experience (the 5th year of a 6-year medical school) have used or believe they will use to select a medical specialty and a residency program [4]. It explores additional aspects of the selection process such as the influence of family and colleagues on specialty and residency decisions, as well as the interests of the students in academic pursuits. It also investigates the hypothesis that finding a specialty interesting and challenging is an overwhelming consideration that negates other important considerations, such as going for residency to a peripherally located hospital (such as in the Galilee in Israel’s north or in the southern area of the country such as Be’er Sheva and Ashkelon) rather than to a hospital in the more densely populated central section such as Tel Aviv or Haifa. Another hypothesis tested was that older students, especially married ones, would be less interested in waiting 2-3 years to begin a residency program in their first-choice specialty. Fifth-year students were studied since our previous study found that most had already begun thinking about a specialty [4]. For the health system leadership to influence specialty decisions, they would benefit from being aware of the students’ thought patterns during the early phases of their decision process.

## Methods

The study spanned 6 years, comprising 6 consecutive 5th-year medical school classes of the Hebrew University – Hadassah School of Medicine. We designed a questionnaire that explored various aspects of the medical specialty selection process. It was based on the results of factor analysis from a previous questionnaire allowing us to reduce the number of original questions while providing space for new ones [4]. The new questions examined the influence of family (i.e. the student’s original family: parents, siblings, etc) and colleagues on specialty and residency decisions, interest in primary care, when the specialty selection process began (i.e. before or during medical school) and interest in academic pursuits. Our previous studies found that the most important criterion for choosing a specialty is that the students find it interesting and challenging [4–6]. In the present study we further examined the definition of a “challenge”, specifically, whether it is more intellectual or physical.

The questionnaire included free-text queries, multiple choice questions and questions with 5-point Likert scales. It elicited (1) demographic information; (2) information on whether the student had already considered a specialty for their residency; which specialty or specialties they were considering (free-text); when they had first considered a specialty (prior to beginning medical school or when during medical school); and whether and when they had changed their mind; (3) the criteria for choosing a career specialty {20 items, 10 new, 5-point Likert scales}; (4) the criteria for choosing a residency program {20 items, 9 new, 5-point Likert scales}; and (5) the importance of finding a specialty interesting and challenging when choosing a residency {3 new items, multiple choice}.

We performed two small (15 students each) pilot studies in order to identify problems and test the user-friendliness of the questionnaire. On the basis of these pilot studies we modified the questionnaires which were then distributed to the 5th year classes of the Hebrew University – Hadassah School of Medicine during the 2010-2011, 2011-2012, 2012-2013, 2013-2014, 2014-2015 and 2015-2016 academic-years.

### Data analysis

Data entered into Excel (Microsoft Inc., Redmond, WA) spreadsheets were analyzed using Excel and Systat 12 (San Jose CA).

The major a priori decision was to compare the four student subgroups enumerated above. In the group admitted to medical school upon completing high school and deferring military service until after their internship, the responses of the students who were admitted on an individual basis to medical school immediately after high school graduation were combined with those participating in the Israel Defense Force’s Medical School Program at the Hebrew University, which is the only one of its kind in Israel.

Another a priori decision was to compare the responses of men and women students. This decision was based on prior research demonstrating significant gender differences associated with specialty selection [4].

After initial data analysis, a *post-hoc* decision was made to compare the responses of married and single students who had completed their army service.

Replies to multiple choice questions are presented as frequency distributions. When Likert Scale responses were considered continuous variables, statistical analyses were performed using all 5 points. When used as categorical variables they were compressed into three categories, (the two points representing negative tendencies and the two points representing positive tendencies were combined). The percentage of responses for each of the three categories (positive tendency, middle point and negative tendency) was then computed.

Continuous variables were compared using two-tailed Student’s t-tests with Bonferroni corrections employed for multiple comparisons. Categorical data are presented as frequency distributions and analyzed using χ^2^ or Fisher exact tests, as appropriate. Backward stepwise multiple variable regression analysis was performed using age as the dependent variable. The independent variables were the specialty and residency selection criteria plus the demographic information. Statistical significance was defined as a *p* value of < 0.05.

The criteria for specialty and residency program selection were subject to hierarchal cluster and factor analyses. In the latter we used varimax rotation with factors having eigenvalues of ≥1.0.

The Institutional Review Board of the Hadassah Medical Organization approved this study. Completion of the questionnaire by the student was considered tacit consent. Participation in this study was voluntary and no incentives were provided to participate.

## Results

Completed questionnaires were returned by 540 of 769 (70%) 5th-year students. Demographic information can be found in Table 1. There was much interest in controllable lifestyle among both genders when choosing both a specialty and a residency program (Tables 1 and 2). There were differences in the decision process between choosing a medical specialty and choosing which residency program to join. Family and colleagues had minimal influence in choosing a specialty while family and their residential locality had much influence on the choice of a residency program, especially among women students (Table 2). Older age was associated with marriage plus spousal influence and time with family as specialty selection criteria. Older age was also associated with family living locale as a criteria for choosing a residency program.

**Table 1:** Demographic and Other Parameters - Men vs Women

		ALL	WOMEN	MEN	*WOMEN* vs *MEN*
	N	540	258	282	
	PERCENT		48.4%	51.6%	
AGE (YEARS)	18-20	0.2%	0.0%	0.4%	
	21-23	16.5%	16.3%	16.7%	
	24-26	28.6%	40.3%	17.4%	
	27-29	40.1%	36.4%	43.5%	
	30-32	10.9%	5.4%	16.3%	
	+ 32	3.7%	1.6%	5.8%	*p < 0.01*
MILITARY SERVICE	COMPLETED OBLIGATION	65.8%	64.5%	67.8%	
	PRE-SERVICE	19.9%	14.5%	24.3%	
	COMPLETED NATIONAL SERVICE	5.6%	10.9%	0.4%	
	NO ARMY SERVICE	8.7%	10.2%	7.6%	*p < 0.05*
MARITAL STATUS	SINGLE	68.5%	69.4%	67.0%	
	MARRIED	30.7%	30.6%	31.5%	
	DIVORCED	0.7%	0.0%	1.4%	*NS*
	STUDENTS WITH CHILDREN	57	23	34	
	TOTAL NUMBER OF CHILDREN	80	28	52	
RELIGIOUS OBSERVANCE	RELIGIOUS	20.1%	19.8%	20.8%	
	SECULAR	66.0%	64.7%	66.4%	
	TRADITIONAL	13.8%	15.1%	12.8%	
HAVE YOU THOUGHT OF OR CONSIDERED A SPECIALTY?	YES	81.5%	83.5%	79.9%	
	NO	18.5%	16.5%	20.1%	*NS*
WHEN DID YOU START CONSIDERING A SPECIALTY?	PRIOR TO BEGINING MEDICAL SCHOOL	27.7%	31.3%	24.2%	
	YEAR 1	3.7%	4.7%	2.8%	
	YEAR 2	2.6%	1.9%	3.3%	
	YEAR 3	4.2%	3.8%	4.7%	
	YEAR 4	46.5%	45.5%	47.4%	
	YEAR 5	15.3%	12.8%	17.7%	*NS*
DID YOU CHANGE YOUR MIND?	YES	60.5%	60.6%	60.2%	
	NO	39.5%	39.4%	39.8%	*NS*
WHEN DID YOU CHANGE YOUR MIND?	YEAR 1	0.8%	0.8%	0.8%	
	YEAR 2	1.2%	0.8%	1.7%	
	YEAR 3	1.6%	1.6%	1.7%	
	YEAR 4	43.3%	42.6%	44.2%	
	YEAR 5	53.1%	54.1%	51.7%	*NS*
SPECIALTIES CONSIDERED BY THE STUDENTS	FAMILY MEDICINE	2.9%	2.9%	2.4%	
	PSYCHIATRY	6.4%	6.6%	6.3%	
	PEDIATRICS	34.9%	42.4%	24.3%	
	INTERNAL MEDICINE	30.2%	26.7%	35.4%	
	OB/GYN	20.4%	27.1%	15.0%	
	EMERGENCY MEDICINE	1.9%	0.0%	3.4%	
	SURGICAL SPECIALTIES	33.7%	27.6%	43.7%	
	OTHER	35.4%	31.9%	39.5%	*p < 0.03*

**Table 2:** Selection Criteria - Men vs Women

	ALL	WOMEN	MEN	*WOMEN* vs *MEN*
N	540	258	282	
*CRITERIA FOR CHOOSING A SPECIALTY* ^*a*^				
TIME WITH FAMILY (1)^b^	69%	**75%**	63%	*p < 0.002*
CONTROLLABLE LIFESTYLE (1)	67%	**72%**	63%	*p < 0.006*
SPECIALTY THAT DEALS WITH SOCIAL ISSUES (3)	30%	**37%**	23%	*p < 0.0001*
DAYTIME WORK ONLY (1)	25%	**28%**	16%	*p < 0.001*
ADVANCING RAPIDLY (2)	60%	55%	**64%**	*p < 0.01*
PROCEDURES/SURGERY	48%	41%	**55%**	*p < 0.0003*
HIGH SALARY	47%	41%	**53%**	*p < 0.005*
PRIVATE PRACTICE	39%	34%	**45%**	*p < 0.007*
OPPORTUNITY FOR RESEARCH (2)	40%	36%	**44%**	*p < 0.01*
ACADEMIC FACULTY MEMBER	27%	23%	**31%**	*p < 0.01*
BEDSIDE SPECIALTY	93%	93%	93%	*NS*
WIDE RANGE OF MEDICAL PROBLEMS	72%	68%	75%	*NS*
INDEPENDENT PRACTICE	55%	54%	57%	*NS*
SPECIALTY WITH TEAMWORK	51%	49%	52%	*NS*
INFLUENCE OF SPOUSE	38%	37%	40%	*NS*
INFLUENCE OF FAMILY	10%	12%	9%	*NS*
WORK ONLY IN THE COMMUNITY	4%	5%	3%	*NS*
NARROW RANGE OF MEDICAL PROBLEMS ©^c^	3%	2%	4%	*NS*
SPECIALTY THAT MY COLEAGUES CHOOSE ©	1%	0%	1%	*NS*
INFLUENCE OF CLASSMATES ©	1%	0%	3%	*NS*
*CRITERIA FOR CHOOSING A RESIDENCY* ^*a*^				
FAMILY LIVING LOCATION	71%	**78%**	65%	*p < 0.002*
CONTROLLABLE LIFESTYLE	65%	**70%**	60%	*p < 0.0001*
SPECIFIC LOCATION	64%	**71%**	58%	*p < 0.005*
MUCH SUPERVISION BY SENIOR PHYSICIANS	43%	**50%**	36%	*p < 0.007*
PRE-DETERMINED WORK HOURS (2)	42%	**47%**	37%	*p < 0.01*
INFLUENCE OF FAMILY	35%	**43%**	28%	*p < 0.001*
LIMITED WORK HOURS	24%	**29%**	18%	*p < 0.02*
MAKE CLINICAL DECISIONS ON YOUR OWN	55%	47%	**62%**	*p < 0.01*
MUCH “ACTION”	42%	39%	**46%**	*p < 0.05*
TEACHING STUDENTS	43%	37%	**48%**	*p < 0.01*
MANY ON-CALL SHIFTS	11%	9%	**13%**	*p < 0.0001*
INTELLECTUAL CHALLENGE (1) ©	83%	84%	82%	*NS*
LEADING DEPARTMENT (1) ©	76%	77%	75%	*NS*
LARGE HOSPTIAL	57%	53%	60%	*NS*
PHYSICAL CHALLENGE	44%	41%	45%	*NS*
OPPORTUNITY FOR RESEARCH	26%	22%	29%	*NS*
PRIMARY CARE	19%	17%	21%	*NS*
SHORT RESIDENCY	15%	17%	14%	*NS*
MUCH CLINIC TIME (2)	12%	12%	11%	*NS*
HOSPITAL IN THE PERIPHERY (3)	9%	8%	9%	*NS*
Residency in a non-leading department with pleasant working conditions	43%	50%	36%	
Residency in a leading department with unpleasant working conditions	57%	50%	64%	*p < 0.05*
Residency in a hospital in the periphery in a specialty that interests me	89%	91%	88%	
Residency in a university hospital in a specialty that interests me less	11%	9%	12%	*NS*
Residency in a specialty that interests me but 2-3 year wait	64%	63%	66%	
Residency in a specialty that interests me less but residency within a year	35%	37%	34%	*NS*

^a^Percent of “agree” and “agree much” responses on 5-point Likert Scale

^b^Numbers in parenthesis are the results of factor analysis

^c^©-cluster per cluster analysis

Boldface entries are the higher value of a significant pair

### Subgroups

Two-thirds of the students had completed military service, 20% were attending medical school prior to military service, 5% had completed national service and 9% had entered medical school without national or military service (Tables 3, 4, 5 and 6).

**Table 3:** SUBGROUPS - DEMOGRAPHIC AND OTHER VARIABLES

		PRE-MILITARY	POST-MILITARY	*PRE* vs *POST MILITARY*	NO MILITARY	*PRE* vs *NO MILITARY*
N		108	356	*p*	47	*p*
	FEMALE	35.6%	46.9%		55.3%	
	MALE	64.4%	53.1%	*p < 0.040*	44.7%	*p < 0.01*
AGE (YEARS)	18-20	0.9%	0.0%		0.0%	
	21-23	67.9%	0.0%		23.4%	
	24-26	30.2%	18.9%		76.6%	
	27-29	0.9%	59.0%		0.0%	
	30-32	0.0%	16.7%		0.0%	
	+ 32	0.0%	5.4%	*p < 0.001*	0.0%	*p < 0.01*
MARITAL STATUS	SINGLE	90.7%	59.9%		95.7%	
	MARRIED	9.3%	39.0%		4.3%	
	DIVORCED	0.0%	1.1%	*p < 0.010*	0.0%	*NS*
CHILDREN	NUMBER	2	44		1	
	TOTAL	2	63		1	
RELIGIOUS OBSERVANCE	RELIGIOUS	12.3%	17.2%		17.4%	
	SECULAR	66.0%	72.9%		50.0%	
	TRADITIONAL	21.7%	9.6%	*NS*	32.6%	*NS*
HAVE YOU THOUGHT OR CONSIDERED A SPECIALTY?	YES	82.2%	81.0%		86.4%	
	NO	17.8%	19.0%	*NS*	13.6%	*NS*
WHEN DID YOU START CONSIDERING A SPECIALTY	PRIOR TO STARTING MEDICAL SCHOOL	23.9%	28.0%		21.6%	
	YEAR 1	4.5%	2.8%		8.1%	
	YEAR 2	2.3%	2.5%		5.4%	
	YEAR 3	5.7%	4.3%		2.7%	
	YEAR 4	52.3%	46.8%		40.5%	
	YEAR 5	11.4%	15.6%	*NS*	21.6%	*NS*
DID YOU CHANGE YOUR MIND?	YES	56.3%	63.1%		59.5%	
	NO	43.7%	36.9%	*p < 0.050*	40.5%	*NS*
WHEN DID YOU CHANGE YOUR MIND?	YEAR 1	2.1%	0.0%		4.8%	
	YEAR 2	0.0%	2.2%		0.0%	
	YEAR 3	2.1%	1.4%		0.0%	
	YEAR 4	48.9%	43.9%		33.3%	
	YEAR 5	46.8%	52.5%	*NS*	61.9%	*p < 0.05*
SPECIALTIES CONSIDERED BY STUDENTS	FAMILY MEDICINE	2.4%	3.2%		2.6%	
	PSYCHIATRY	4.8%	10.5%		2.6%	
	PEDIATRICS	34.5%	34.3%		28.9%	
	INTERNAL MEDICINE	31.0%	30.0%		23.7%	
	OB/GYN	15.5%	20.6%		31.6%	
	EMERGENCY MEDICINE	1.2%	1.4%		5.3%	
	SURGICAL SPECIALTIES	33.3%	33.6%		26.3%	
	OTHER	36.9%	33.2%	*NS*	31.6%	*NS*

**Table 4:** SUBGROUPS SELECTION CRITERIA

	PRE-MILITARY	POST-MILITARY	*PRE* vs *POST MILITARY*	NO MILITARY	*PRE* vs *NO MILITARY*
N	108	356	*p*	47	*p*
*CRITERIA FOR CHOOSING A SPECIALTY* ^*a*^					
ACADEMIC FACULTY MEMBER	**33.0%**	22.1%	*p < 0.040*	**47.8%**	*p < 0.02*
TIME WITH FAMILY	19.6%	**31.8%**	*p < 0.010*	**30.4%**	*p < 0.05*
BEDSIDE SPECIALTY	90.7%	93.2%	*NS*	89.4%	*NS*
WIDE RANGE OF MEDICAL PROBLEMS	74.8%	71.3%	*NS*	61.7%	*NS*
TIME WITH FAMILY (1)^b^	65.4%	71.5%	*NS*	60.9%	*NS*
CONTROLLABLE LIFESTYLE (1)	65.1%	68.4%	*NS*	72.3%	*NS*
ADVANCING RAPIDLY (2)	60.7%	59.2%	*NS*	41.8%	*NS*
INDEPENDENT PRACTICE	57.9%	53.1%	*NS*	***69.6%***	*p < 0.01*
PROCEDURES/SURGERY	50.9%	47.9%	*NS*	46.8%	*NS*
HIGH SALARY	49.1%	46.6%	*NS*	74.5%	*NS*
SPECIALTY WITH TEAMWORK	47.7%	53.1%	*NS*	46.8%	*NS*
OPPORTUNITY FOR RESEARCH (2)	42.5%	35.3%	*NS*	***55.3%***	*p < 0.03*
PRIVATE PRACTICE	40.0%	39.9%	*NS*	42.6%	*NS*
INFLUENCE OF SPOUSE	***35.5%***	40.9%	*NS*	25.0%	*p < 0.04*
DAYTIME WORK ONLY (1)	18.9%	22.2%	*NS*	26.7%	*NS*
INFLUENCE OF FAMILY	11.4%	9.9%	*NS*	12.8%	*NS*
WORK ONLY IN THE COMMUNITY	5.6%	3.7%	*NS*	4.3%	*NS*
NARROW RANGE OF MEDICAL PROBLEMS ©^c^	2.8%	3.1%	*NS*	4.3%	*NS*
INFLUENCE OF CLASSMATES©	1.9%	1.1%	*NS*	4.3%	*NS*
SPECIALTY THAT MY COLEAGUES CHOOSE©	0.9%	1.1%	*NS*	0.0%	*NS*
*CRITERIA FOR CHOOSING A RESIDENCY* ^*a*^					
LEADING DEPARTMENT (1)©	**85.0%**	71.8%	*p < 0.002*	83.0%	*NS*
TEACHING STUDENTS	**53.3%**	39.1%	*p < 0.004*	59.6%	*NS*
OPPORTUNITY FOR RESEARCH	**27.1%**	22.9%	*p < 0.040*	**40.4%**	*p < 0.04*
MANY ON-CALL SHIFTS	**17.8%**	8.8%	*p < 0.008*	17.0%	*NS*
FAMILY LIVING LOCATION	64.2%	**76.3%**	*p < 0.006*	53.2%	*NS*
SPECIFIC LOCATION	49.5%	**69.0%**	*p < 0.006*	63.8%	*p < 0.05*
INFLUENCE OF FAMILY	21.7%	**38.7%**	*p < 0.020*	**40.4%**	*p < 0.04*
INTELLECTUAL CHALLENGE (1)©	85.0%	82.5%	*NS*	83.0%	*NS*
CONTROLLABLE LIFESTYLE	64.5%	64.3%	*NS*	**74.5%**	*p < 0.03*
MAKE CLINICAL DECISIONS ON YOUR OWN	59.8%	55.9%	*NS*	46.8%	*p < 0.05*
LARGE HOSPTIAL	58.9%	54.1%	*NS*	70.2%	*NS*
MUCH “ACTION”	46.2%	41.0%	*NS*	53.2%	*NS*
PHYSICAL CHALLENGE	45.3%	43.5%	*NS*	48.9%	*NS*
PRE-DETERMINED WORK HOURS (2)	44.9%	40.1%	*NS*	42.6%	*NS*
MUCH SUPERVISION BY SENIOR PHYSICIANS	42.1%	42.2%	*NS*	38.3%	*NS*
PRIMARY CARE	21.7%	19.3%	*NS*	19.6%	*NS*
LIMITED WORK HOURS	21.5%	23.9%	*NS*	23.4%	*NS*
SHORT RESIDENCY	20.8%	13.6%	*NS*	17.0%	*NS*
MUCH CLINIC TIME (2)	11.3%	11.3%	*NS*	14.9%	*NS*
HOSPITAL IN THE PERIPHERY (3)	10.3%	8.8%	*NS*	8.5%	*NS*
Residency in a non-leading department with pleasant working conditions	30.2%	49.7%		21.7%	
Residency in a leading department with unpleasant working conditions	69.8%	50.3%	*p < 0.010*	78.3%	*NS*
Residency in a hopsital in the periphery in a specialty that interests me	91.6%	88.9%		89.1%	
Residency in a university hopsital in a specialty that interests me less	8.4%	11.1%	*NS*	10.9%	*NS*
Residency in a specialty that interests me but 2-3 year wait	75.7%	60.3%		61.7%	
Residency in a specialty that interests me less but residency within a year	24.3%	39.7%	*p < 0.010*	38.3%	*p < 0.03*

^a^Percent of “agree” and “agree much” responses on 5-point Likert Scale

^b^Numbers in parenthesis are the results of factor analysis

^c^© - clusters per cluster analysis

Boldface entries are the higher value of a significant pair

**Table 5:** Comparison of Subgroups - Demographic and Other Information

		PRE-MILITARY	PRE- MILITARY	PRE- MILITARY	POST- MILITARY	POST-MILITARY	POST-MILITARY	*PRE-MILITARY* vs *POST-MILITARY*		NATIONAL SERVICE	*NATIONAL SERVICE* vs *POST-MILITARY*
		FEMALE	MALE	*m-*vs*-f*	FEMALE	MALE	*m-*vs*-f*	*f*	*m*		*FEMALE*
N		38	70	*p*	165	189	*p*	*p*	*p*	29	*p*
FEMALE										**96.55%**	
AGE (YEARS)	18-20	0.0%	1.4%		0.0%	0.0%				0.0%	
	21-23	73.0%	65.2%		0.0%	0.0%				20.7%	
	24-26	27.0%	31.9%		36.4%	3.7%				55.2%	
	27-29	0.0%	1.4%		53.3%	64.0%				20.7%	
	30-32	0.0%	0.0%		8.5%	23.8%				0.0%	
	+ 32	0.0%	0.0%	*NS*	1.8%	8.5%	*0.05*	*p < 0.01*	*p < 0.01*	3.4%	*p < 0.001*
MARITAL STATUS	SINGLE	89.2%	91.4%		66.1%	54.5%				41.4%	
	MARRIED	10.8%	8.6%		33.9%	43.4%				58.6%	
	DIVORCED	0.0%	0.0%	*NS*	0.0%	2.1%	*NS*	*p < 0.001*	*p < 0.001*	0.0%	*p < 0.001*
CHILDREN	NUMBER	0	2		12	32				10	
	TOTAL	0	2		13	50				14	
RELIGIOUS OBSERVANCE	RELIGIOUS	8.1%	14.5%		9.7%	23.8%				86.2%	
	SECULAR	70.3%	63.8%		78.2%	68.3%				10.3%	
	TRADITIONAL	21.6%	21.7%	*NS*	11.5%	7.9%	*p < 0.05*	*p < 0.05*	*p < 0.05*	3.4%	*p < 0.001*
HAVE YOU THOUGHT OR CONSIDERED A SPECIALTY?	YES	91.9%	77.1%		81.7%	80.3%				82.8%	
	NO	8.1%	22.9%	*p < 0.05*	18.3%	19.7%	*NS*	*NS*	*NS*	17.2%	*NS*
WHEN DID YOU START CONSIDERING A SPECIALTY?	PRIOR	23.5%	24.1%		30.8%	25.5%				47.8%	
	YEAR 1	5.9%	3.7%		3.8%	2.0%				4.3%	
	YEAR 2	2.9%	1.9%		0.8%	4.0%				0.0%	
	YEAR 3	5.9%	5.6%		3.8%	4.7%				0.0%	
	YEAR 4	52.9%	51.9%		47.4%	46.3%				34.8%	
	YEAR 5	8.8%	13.0%	*NS*	13.5%	17.4%	*NS*	*NS*	*NS*	13.0%	*p < 0.04*
DID YOU CHANGE YOUR MIND?	YES	52.9%	58.5%		65.5%	61.0%				56.5%	
	NO	47.1%	41.5%	*NS*	34.5%	39.0%	*NS*	*NS*	*NS*	43.5%	*NS*
WHEN DID YOU CHANGE YOUR MIND?	YEAR 1	0.0%	3.4%		0.0%	0.0%				0.0%	
	YEAR 2	0.0%	0.0%		1.4%	2.9%				0.0%	
	YEAR 3	5.6%	0.0%		1.4%	1.4%				0.0%	
	YEAR 4	38.9%	55.2%		43.5%	44.3%				53.8%	
	YEAR 5	55.6%	41.4%	*NS*	53.6%	51.4%	*NS*	*NS*	*NS*	46.2%	*NS*
SPECIALTIES CONSIDERED BY STUDENTS	FAMILY MEDICINE	0.0%	3.9%		4.5%	2.1%				0.0%	
	PSYCHIATRY	6.1%	5.9%		12.0%	6.3%				8.3%	
	PEDIATRICS	36.4%	27.5%		42.1%	25.2%				45.8%	
	INTERNAL MEDICINE	24.2%	37.3%		27.8%	32.9%				37.5%	
	OB/GYN	21.2%	13.7%		24.1%	17.5%				25.0%	
	EMERGENCY MEDICINE	0.0%	2.0%		0.0%	2.1%				0.0%	
	SURGICAL SPECIALTIES	30.3%	39.2%		27.8%	44.1%				25.0%	
	OTHER	21.2%	39.2%	*p < 0.05*	30.8%	32.2%	*p < 0.02*	*NS*	*NS*	54.2%	*NS*

**Table 6:** Comparison of Subgroups - Selection Criteria

	PRE-MILITARY	PRE-MILITARY	*PRE-MILITARY*	POST-MILITARY	POST-MILITARY	*POST-MILITARY*	*PRE-MILITARY* vs *POST-MILITARY*		NATIONAL SERVICE	*NATIONAL SERVICE* vs *POST-MILITARY*
	FEMALE	MALE	*m-*vs*-f*	FEMALE	MALE	*m-*vs*-f*	*f*	*m*		*FEMALE*
N	38	70	*p*	165	189	*p*	*p*	*p*	29	*p*
*CRITERIA FOR CHOOSING A SPECIALTY*										
TIME WITH FAMILY	70.3%	62.9%	*NS*	**79.4%**	64.6%	*p < 0.009*	*NS*	*NS*	62.1%	*p < 0.05*
CONTROLLABLE LIFESTYLE	70.3%	62.9%	*NS*	**74.5%**	63.0%	*p < 0.002*	*NS*	*NS*	51.7%	*p < 0.05*
SPECIALTY DEALING WITH SOCIAL ISSUES	27.0%	15.7%	*NS*	**37.8%**	26.6%	*p < 0.003*	*0.05*	*NS*	51.7%	*p < 0.03*
DAYTIME WORK ONLY	**29.7%**	14.3%	*p < 0.05*	**26.7%**	18.2%	*p < 0.05*	*NS*	*NS*	27.6%	*NS*
PROCEDURES/SURGERY	48.6%	51.4%	*NS*	38.2%	**56.4%**	*p < 0.0001*	*NS*	*NS*	39.3%	*NS*
HIGH SALARY	51.4%	48.6%	*NS*	39.0%	**53.2%**	*p < 0.02*	*NS*	*NS*	34.5%	*NS*
PRIVATE PRACTICE	38.9%	40.6%	*NS*	31.7%	**47.1%**	*p < 0.004*	*NS*	*NS*	24.1%	*NS*
OPPORTUNITY FOR RESEARCH	36.1%	45.7%	*NS*	29.7%	**40.2%**	*p < 0.008*	*NS*	*NS*	58.6%	*p < 0.04*
BEDSIDE SPECIALTY	91.9%	90.0%	*NS*	90.2%	95.7%	*NS*	*NS*	*NS*	100.0%	*NS*
WIDE RANGE OF MEDICAL PROBLEMS	67.6%	78.6%	*NS*	67.7%	74.5%	*NS*	*NS*	*NS*	69.0%	*NS*
ADVANCING RAPIDLY	51.4%	65.7%	*NS*	55.8%	62.2%	*NS*	*NS*	*NS*	58.6%	*NS*
SPECIALTY WITH TEAMWORK	51.4%	45.7%	*NS*	51.5%	54.5%	*NS*	*NS*	*NS*	41.4%	*NS*
INDEPENDENT PRACTICE	62.2%	55.7%	*NS*	49.7%	56.1%	*NS*	*NS*	*NS*	51.7%	*NS*
INFLUENCY OF SPOUSE	35.1%	35.7%	*NS*	38.4%	40.2%	*NS*	*NS*	*NS*	35.7%	*NS*
SPECIALTY THAT MY COLEAGUES CHOOSE	0.0%	1.4%	*NS*	0.6%	1.6%	*NS*	*NS*	*NS*	0.0%	*NS*
ACADEMIC FACULTY MEMBER	24.3%	37.1%	*NS*	19.4%	24.5%	*NS*	*NS*	*p < 0.03*	27.6%	*NS*
INFLUENCE OF FAMILY	10.8%	11.8%	*NS*	12.2%	7.9%	*NS*	*NS*	*NS*	6.9%	*NS*
WORK ONLY IN THE COMMUNITY	5.4%	5.7%	*NS*	4.9%	2.6%	*NS*	*NS*	*NS*	3.4%	*NS*
NARROW RANGE OF MEDICAL PROBLEMS	0.0%	4.3%	*NS*	3.0%	3.2%	*NS*	*NS*	*NS*	3.4%	*NS*
INFLUENCE OF CLASSMATE	0.0%	2.9%	*NS*	0.0%	2.1%	*NS*	*NS*	*NS*	0.0%	*NS*
*CRITERIA FOR CHOOSING A RESIDENCY*										
FAMILY LIVING LOCATION	67.6%	62.3%	*NS*	**83.6%**	69.8%	*< 0.003*	*p < 0.03*	*NS*	55.2%	*p < 0.03*
SPECIFIC LOCATION	51.4%	48.6%	*NS*	**76.8%**	62.2%	*p < 0.01*	*p < 0.03*	*NS*	60.7%	*NS*
CONTROLLABLE LIFESTYLE	67.6%	62.9%	*NS*	**72.0%**	57.7%	*p < 0.0002*	*NS*	*NS*	58.6%	*NS*
PRE-DETERMINED WORK HOURS	54.1%	40.0%	*NS*	**46.1%**	34.9%	*p < 0.01*	*NS*	*NS*	51.7%	*NS*
INFLUENCE OF FAMILY	19.4%	22.9%	*NS*	**47.9%**	30.7%	*p < 0.0006*	*p < 0.001*	*NS*	34.5%	*NS*
MAKE CLINICAL DECISIONS ON YOUR OWN	62.2%	58.6%	*NS*	46.1%	**64.6%**	*p < 0.002*	*p < 0.03*	*0.05*	37.9%	*p < 0.05*
OPPORTUNITY FOR RESEARCH	18.9%	31.4%	*0.02*	18.8%	**26.5%**	*p < 0.05*	*NS*	*NS*	31.0%	*p < 0.04*
INTELLECTUAL CHALLENGE	83.8%	85.7%	*NS*	86.7%	78.8%	*NS*	*NS*	*NS*	79.3%	*NS*
LEADING DEPARTMENT	81.1%	87.1%	*NS*	74.5%	69.3%	*NS*	*NS*	*p < 0.01*	79.3%	*NS*
LARGE HOSPTIAL	45.9%	65.7%	*NS*	51.2%	56.4%	*NS*	*NS*	*NS*	58.6%	*NS*
MUCH SUPERVISION BY SENIOR PHYSICIANS	54.1%	35.7%	*0.05*	47.3%	37.8%	*NS*	*NS*	*NS*	62.1%	*p < 0.04*
PHYSICAL CHALLENGE	45.9%	44.9%	*NS*	41.2%	45.5%	*NS*	*NS*	*NS*	27.6%	*p < 0.04*
MUCH “ACTION”	50.0%	44.3%	*NS*	37.0%	44.4%	*NS*	*NS*	*NS*	20.7%	*p < 0.05*
TEACHING STUDENTS	37.8%	61.4%	*0.008*	35.4%	42.3%	*NS*	*NS*	*p < 0.0005*	34.5%	*NS*
LIMITED WORK HOURS	27.0%	18.6%	*NS*	29.3%	19.3%	*NS*	*NS*	*NS*	24.1%	*NS*
SHORT RESIDENCY	24.3%	18.8%	*NS*	15.2%	12.2%	*NS*	*NS*	*NS*	17.2%	*NS*
PRIMARY CARE	21.6%	21.7%	*NS*	16.0%	22.2%	*NS*	*NS*	*NS*	8.0%	*NS*
MUCH CLINIC TIME	8.3%	12.9%	*NS*	11.5%	11.1%	*NS*	*NS*	*NS*	10.3%	*NS*
PERIPHERAL HOSPITAL	8.1%	11.4%	*NS*	8.5%	9.0%	*NS*	*NS*	*NS*	6.9%	*NS*
MANY ON-CALL SHIFTS	13.5%	20.0%	*0.04*	7.9%	9.5%	*NS*	*NS*	*p < 0.02*	6.9%	*NS*
Residency in a non-leading department with pleasant working conditions	35.1%	27.5%		57.6%	41.9%				37.9%	
Residency in a leading department with unpleasant working conditions	64.9%	72.5%	*NS*	42.4%	58.1%	*p < 0.01*	*p < 0.01*	*0.05*	62.1%	*p < 0.002*
Residency in a hopsital in the periphery in a specialty that interests me	91.9%	91.4%		90.7%	87.2%				86.2%	
Residency in a university hopsital in a specialty that interests me less	8.1%	8.6%	*NS*	9.3%	12.8%	*NS*	*NS*	*NS*	13.8%	*NS*
Residency in a specialty that interests me but 2-3 year wait	78.4%	74.3%		57.8%	62.6%				71.4%	
Residency in a specialty that interests me less but residency within a year	21.6%	25.7%	*NS*	42.2%	37.4%	*NS*	*p < 0.05*	*NS*	28.6%	*NS*

Boldface entries are the higher value of a significant pair

#### Gender

There were differences in the gender distribution between the various groups. There were a greater proportion of women in the group that had completed military service (47%) than among those attending medical school prior to military service (36%). The influence of gender was further delineated by comparing the replies of the male and female medical students in these two groups (Tables 5 and 6).

#### Age

To provide a similar age basis for comparison we compared the group that did not perform military to the pre-military service group (Tables 3 and 4), and to provide similar age and gender comparisons the group that had completed national service (97% women) was compared with the women students who had completed military service (Tables 5 and 6).

#### Family status

Over a third (39%) of the students who had completed military service were married, providing an opportunity to compare the responses of married and unmarried students (Tables 7 and 8).

**Table 7:** Single vs Married Students who Completed Military Service - Demographics

		SINGLE	MARRIED	*P*
N		212	142	
FEMALE		51.7%	39.7%	
MALE		48.3%	60.3%	*0.05*
AGE (YEARS)	18-20	0.0%	0.0%	
	21-23	0.0%	0.0%	
	24-26	24.5%	10.6%	
	27-29	60.4%	57.0%	
	30-32	10.4%	26.1%	
	+ 32	4.7%	6.3%	*0.01*
RELIGIOUS OBSERVANCE	RELIGIOUS	6.6%	33.1%	
	SECULAR	81.1%	60.6%	
	TRADITIONAL	12.3%	5.6%	*0.01*
THOUGHT OR CONSIDERED A SPECIALTY?	YES	81.1%	80.7%	
	NO	18.9%	19.3%	*NS*
WHEN DID YOU START CONSIDERING A SPECIALTY?	PRIOR TO STARTING MEDICAL SCHOOL	29.4%	25.9%	
	YEAR 1	4.7%	0.0%	
	YEAR 2	2.9%	1.8%	
	YEAR 3	4.1%	4.5%	
	YEAR 4	44.1%	50.9%	
	YEAR 5	14.7%	17.0%	*NS*
DID YOU CHANGE YOUR MIND?	YES	64.5%	61.1%	
	NO	35.5%	38.9%	*NS*
WHEN DID YOU CHANGE YOUR MIND?	YEAR 1	0.0%	0.0%	
	YEAR 2	1.2%	3.6%	
	YEAR 3	2.4%	0.0%	
	YEAR 4	48.8%	36.4%	
	YEAR 5	47.6%	60.0%	*NS*
SPECIALTIES CONSIDERED BY THE STUDENTS	FAMILY MEDICINE	3.6%	2.8%	
	PSYCHIATRY	10.1%	8.4%	
	PEDIATRICS	34.9%	34.6%	
	INTERNAL MEDICINE	27.8%	30.8%	
	OB/GYN	21.9%	16.8%	
	EMERGENCY MEDICINE	1.8%	0.9%	
	SURGICAL SPECIALTIES	39.1%	30.8%	
	OTHER	31.4%	36.4%	*NS*

**Table 8:** Single vs Married Students who Completed Military Service - Selection Criteria

	SINGLE	MARRIED	*P*
N	212	142	
*CRITERIA FOR CHOOSING A SPECIALTY* ^*a*^			
INFLUENCE OF SPOUSE	30.8%	**56.0%**	*p < 0.001*
BEDSIDE SPECIALTY	94.3%	92.9%	*NS*
WIDE RANGE OF MEDICAL PROBLEMS	70.5%	72.5%	*NS*
TIME WITH FAMILY (1)^b^	68.9%	75.4%	*NS*
CONTROLLABLE LIFESTYLE (1)	67.5%	69.7%	*NS*
ADVANCING RAPIDLY (2)	57.8%	61.3%	*NS*
SPECIALTY WITH TEAMWORK	55.7%	49.3%	*NS*
INDEPENDENT PRACTICE	53.8%	52.1%	*NS*
PROCEDURES/SURGERY	45.3%	51.8%	*NS*
HIGH SALARY	44.8%	49.3%	*NS*
PRIVATE PRACTICE	43.6%	34.5%	*NS*
OPPORTUNITY FOR RESEARCH (2)	34.0%	37.3%	*NS*
SPECIALTY THAT DEALS WITH SOCIAL ISSUES (3)	32.7%	30.5%	*NS*
DAYTIME WORK ONLY (1)	22.3%	22.0%	*NS*
ACADEMIC FACULTY MEMBER	20.3%	24.8%	*NS*
INFLUENCE OF FAMILY	8.5%	12.0%	*NS*
WORK ONLY IN THE COMMUNITY	3.8%	3.5%	*NS*
NARROW RANGE OF MEDICAL PROBLEMS ©^c^	3.3%	2.8%	*NS*
INFLUENCE OF CLASSMATES ©	1.4%	0.7%	*NS*
SPECIALTY THAT MY COLEAGUES CHOOSE ©	0.5%	2.1%	*NS*
*CRITERIA FOR CHOOSING A RESIDENCY* ^*a*^			
PHYSICAL CHALLENGE	**49.1%**	35.2%	*p < 0.02*
FAMILY LIVING LOCATION	72.2%	**82.4%**	*p < 0.002*
INFLUENCE OF FAMILY	33.5%	**46.5%**	*p < 0.007*
MUCH CLINIC TIME (2)	8.0%	**16.2%**	*p < 0.05*
INTELLECTUAL CHALLENGE (1) ©	81.6%	83.8%	*NS*
LEADING DEPARTMENT (1) ©	73.1%	69.7%	*NS*
SPECIFIC LOCATION	68.2%	70.2%	*NS*
CONTROLLABLE LIFESTYLE	61.1%	69.0%	*NS*
MAKE CLINICAL DECISIONS ON YOUR OWN	55.2%	57.0%	*NS*
LARGE HOSPTIAL	52.4%	56.7%	*NS*
MUCH SUPERVISION BY SENIOR PHYSICIANS	44.1%	39.4%	*NS*
MUCH “ACTION”	42.9%	38.0%	*NS*
TEACHING STUDENTS	41.5%	35.5%	*NS*
PRE-DETERMINED WORK HOURS (2)	37.3%	44.4%	*NS*
LIMITED WORK HOURS	25.2%	22.0%	*NS*
OPPORTUNITY FOR RESEARCH	19.8%	27.5%	*NS*
PRIMARY CARE	19.3%	19.3%	*NS*
SHORT RESIDENCY	14.6%	12.0%	*NS*
HOSPITAL IN THE PERIPHERY (3)	7.5%	10.6%	*NS*
MANY ON-CALL SHIFTS	9.9%	7.0%	*NS*
Residency in a non-leading department with pleasant working conditions	50.0%	49.3%	
Residency in a leading department with unpleasant working conditions	50.0%	50.7%	*NS*
Residency in a hopsital in the periphery in a specialty that interests me	89.5%	87.9%	
Residency in a university hopsital in a specialty that interests me less	10.5%	12.1%	*NS*
Residency in a specialty that interests me but 2-3 year wait	**64.7%**	53.7%	
Residency in a specialty that interests me less but residency within a year	**35.3%**	46.3%	*p < 0.03*

^a^Percent of “agree” and “agree much” responses on 5-point Likert Scale

^b^Numbers in parenthesis are the results of factor analysis

^c^© - clusters per cluster analysis

Boldface entries are the higher value of a significant pair

## Discussion

Choosing a specialty and choosing a residency program involve different considerations. Choosing a specialty is akin to choosing a career. It requires introspection involving analyzing one’s interests, aptitudes, skills and personality. The results of this analysis then must align with the nature of the specialty [7, 8]. Therefore, choosing a specialty is basically a personal decision as shown by the results of the current study where there was little influence of colleagues or family on the choice. Overall, only one-third of the students replied that their spouse was important in their decision. Moreover, when we examined the students who had completed army service 56% of the married students and 31% of the unmarried ones replied that their spouse was or would be important in their decision. Other studies showed that a joint decision is especially common in two-career families [9]. In contrast, the students reported that choosing a residency program includes family influence, especially as it relates to the location of their family’s residence, an observation that has similarly been made by other investigators [10]. In the present study family influence and location were more important for female than male students. Among students who had completed military service, family residential location was more important for married than single students likely because of their need for assistance and support from other family members. These observations are similar to those made by other investigators [11]. In addition, a spouse’s desire for a career of his/her own and a good educational system for their children limits choice of residency programs and ultimately, practice locations [9]. Spouses of American applicants to thoracic surgery residencies reported that the top 3 factors in choosing a program were the quality of the fellowship, its geographic location and proximity to family [12]. Of the applicants to US emergency medicine residencies 75% rated “preference for a particular geographic location” and 60% “to be close to spouse, significant other, or family” as major criteria for selecting a particular residency program [13]. These differences in the decision processes between selecting a medical specialty and a residency program have import when counseling students. Moreover, the findings of the present study reinforce that involvement of spouses in selecting a residency program and its location should be considered when advising students, especially older and married ones [14].

Our previous studies revealed that the most important criterion for selecting a specialty is that the individual student finds it interesting and challenging [4–6]. The results of the present study further define challenges as being more intellectual than physical. Others have also noted that an intellectual challenge is an important criterion whether selecting a medical or surgical specialty [1, 15]. Furthermore, the current study shows that interest in a specialty is an overwhelming criterion with those students who would rather choose a residency in a peripheral hospital in the specialty that interests them most than choose a residency in a leading hospital in another specialty. This result contravenes the fact that less than 10% of the students answered that they would consider a residency in a peripheral hospital, further demonstrating the tremendous importance of finding a specialty interesting and challenging. Overall, the students also overwhelmingly indicated that they were willing to wait 2-3 years to begin a residency in a specialty that greatly interests them instead of immediately starting a residency in one that interests them less. However, as discussed below, fewer post- than pre-military women reported that they were willing to wait 2-3 years for a residency in the specialty that interested them most and were willing to begin residency immediately after internship in a specialty that interested them less. The observation that many students were willing to wait 2-3 years to begin a residency in a specialty that greatly interests them points to the reason it is often difficult to convince students to change their specialty choices despite there being a lack of residency positions in their preferred specialty and unfilled positions in other specialties [16]. It also helps explain the phenomenon of students waiting 2-4 years for residency positions in especially competitive specialties such as otolaryngology, dermatology, plastic surgery and ophthalmology. The challenge for the healthcare system leadership is how to reduce this backlog of students waiting for residencies in often oversubscribed specialties and channel them to specialties with open positions.

### Subgroups

There were many similarities, as well as some significant differences between the two major subgroups, the pre- and post-military subgroups. Interestingly, despite the pre-military subgroup being younger and facing another year of medical school, a year of internship and at least 5 years of military service, there were no differences between whether and when they had begun to think of which specialty to choose as a career, as compared to the group that had completed military service. This observation likely signifies the central importance of choosing a medical specialty among medical students. The only significant difference was that fewer of the pre-military students had changed their minds about a specialty by the fifth-year.

Among the pre-military students there were more men than women, while in the post-military group there were equal numbers of men and women. To account for the effects of gender we compared the male and female students between the groups. This comparison revealed few differences among the specialty selection criteria between men and women. The exceptions were that more post-military women than men were interested in a specialty that includes dealing with the psycho-social issues confronting their patients while more pre- than post-military men were interested in being members of an academic faculty. This interest in academic pursuits was further demonstrated by pre-military male students being more interested than pre-military females in a residency that includes teaching medical students. The reason for the post-military students being less interested in academic careers might be ascribed to their being 3-4 years older and more likely to be married. They, thus, would desire a secure position upon completing residency when they are already in their early to middle 30’s instead of a position whose advancement is based on the uncertainties of academia, such as the trials and tribulations associated with being a researcher. A similar reduced interest in academic pursuits was found among older Australian medical school graduates [17]. This result is important for Israeli academic medicine, since the younger students, including those in the group that had not performed military service, were more enthusiastic about academic pursuits. Therefore, the healthcare system should identify promising academics, including those in the pre-military program, and channel them into physician-scientist programs. In the case of those in the pre-military program this could include research on military related health subjects. Alternately, this might be ascribed to the determined nature of the pre-military students who were ambitiously embarking on a military career. Whether these academic interests are sustained until the pre-military students finished their military obligations and begin their residencies, should be explored in subsequent studies.

There were differences among the residency program selection criteria between the pre and post-military groups. Post-military women students were more influenced by their families and the location of their families than their pre-military counterparts. These differences might be ascribed to the older and more often married post-military women having children or wanting to begin families. This difference was reinforced by significantly fewer post- than pre-military women reporting that they were willing to wait 2-3 years for a residency in the specialty that interested them most and were willing to begin residency immediately after internship in a specialty that interested them less. This result is comparable to older New Zealand medical students being more likely than younger ones to become general practitioners, thus not opting for specialty training [18]. In many countries without a military obligation, students begin 6-year medical school programs immediately following high school and thus are similar in age to those in the Israeli pre-military program. In many of these schools there are a few older students who are similar in age to those in the post-military group. Therefore, the comparisons between the pre and post military groups might help direct the leadership of 6-year medical schools in countries without a military obligation to compare the career interests and goals of younger and older students. For example, differences between the two groups was further demonstrated when significantly more post-military, than pre-military, women and men replied they would choose a residency in a non-leading department with easier working conditions rather than a leading department with tougher working conditions. Not surprisingly, fewer post-military than pre-military students wanted a residency with many on-calls. These results likely reflect the effects of age, marital status, personal circumstance and the need to balance free time with career demands on decreasing enthusiasm for extended workhours as family obligations become more important [19].

The subgroup that included students who had performed national service was particularly distinctive. It was comprised almost exclusively of religious women. Forty-three percent, as opposed to about 20% in the other groups, had thought of a specialty prior to beginning medical school. This might reflect their exposure to the Israeli healthcare system during their national service since those performing such service have a wide choice of venues, including hospitals and other healthcare facilities. When working in such facilities they often function as nurse’s aides and laboratory assistants. This healthcare exposure might also explain their greater interests in research opportunities and residencies in a leading department with unpleasant working conditions when compared to the women students who had completed military service.

Students in the pre-military program present an interesting challenge since they had already begun thinking about specialties but will only need to definitively decide in another 7 years. This is due to their need to complete their 6th-year of medical school and a year of rotating internship plus a 5-year military obligation. This situation provides a future opportunity to research the effect of serving as military general medical officers for a number of years has on choosing a medical specialty. Namely, whether their medical specialty and residency program selection criteria profiles align with those of the students who had completed their military service before beginning medical school. The 6-year Israeli military medical school program, being part of a civilian university medical school, differs from the 4-year US program of the United States Uniformed Services University Medical School [20]. Additionally, the US program admits students after four years of college and most graduates enter residencies in military hospitals immediately following graduation. This makes it difficult to extrapolate from the US to the Israeli program [21].

### Strengths and limitations

A major strength of this study is the large number of students studied. This permitted us to compare various subgroups. A limitation was that the study was performed in only one of the five Israeli medical schools. However, this medical school includes the only military medical school program in Israel thus providing us with the unique opportunity to compare students who had completed with those who had not yet started military service, as well as the effects of age and marriage on the specialty and residency program selection criteria of these medical students.

## Conclusions

The present study explored medical student’s attitudes as they begin to examine their future professional careers. Over 80% had begun to think about a medical specialty, with the majority starting during their 4th and 5th years of medical school. Many had already changed their choices. Ashkenazi et al. [10] found that 74% of Israeli medical school graduates had decided on their specialty during their clinical years at medical school or during their internship. Therefore, the 5th year appears to be an opportune time for medical schools to begin career counseling and for department chairs and residency program directors to start marketing their specialties and residency programs [22]. The observations made in this study have import beyond Israel. Notably, the importance of differentiating between choosing a specialty and choosing a residency program. Selecting a specialty is a uniquely personal decision with some spousal influence among married students. However, the selection of a residency program and its location involves wider family considerations. Furthermore, this study demonstrates the importance of recognizing that medical school classes are heterogeneous; composed of various subgroups of students. Namely, that the importance of various specialty and residency program selection criteria may differ between the subgroups. These observations should be taken into consideration when counseling and aiding students in making these two important decisions: which specialty to select and which residency program to choose [23]. These observations are also important for department chairs and residency program directors, specifically, that they might have to use different strategies when marketing their specialties and programs to members of various subgroups. For example, marketing specialties and residency programs to older, married students with children might place greater emphasis on being able to positively balance family life with career demands than when marketing specialties and residency programs to younger unmarried students where academic opportunities might be emphasized. The interest in research expressed among both the students who had performed national service and those who had not performed either military or national services is another example of the differences between the subgroups that could also be exploited in marketing campaigns.
